# Exploratory Study on the Challenges of Newborn Screening for Lysosomal Storage Disorders Emphasizes the Need for Multitier Testing and Collaborative Approaches to Management

**DOI:** 10.1002/jmd2.70027

**Published:** 2025-06-16

**Authors:** A. Terrell, K. Sapp, B. Graham, M. McPheron, L. Wetherill

**Affiliations:** ^1^ Department of Medical and Molecular Genetics Indiana University School of Medicine Indianapolis Indiana USA; ^2^ Indiana University Health Indianapolis Indiana USA

**Keywords:** challenges, collaboration, interpretation of results, lysosomal storage disorder, multitier test, newborn screen

## Abstract

Innovative treatments have allowed the introduction of conditions such as lysosomal storage disorders (LSDs) to newborn screening (NBS). This study explored the challenges healthcare providers faced with the addition of LSDs to NBS and identified adjustments that minimized the burden of such challenges. An online survey was distributed to healthcare providers with experience working with patients with LSDs. The most common *anticipated* challenges were interpreting NBS results (75%) and having adequate screening protocols (63%). *After the addition of LSDs*, interpretation of newborn screen results (64%) remained a challenge, but adequate screening protocols were less frequent (25%). Collaboration of care with additional subspecialty providers was the most common change in clinic structure (68%) and individual practice (54%) after the addition of LSDs to NBS. Given the interpretation of results remained a challenge most providers faced, we advocate the implementation of multitier screening protocols is key to improving sensitivity and specificity of NBS for LSDs. This allows for the identification of at‐risk infants and provides clarity on expected phenotypes and healthcare needs. These results indicate collaboration between healthcare providers is a key factor in providing optimal care. The findings of this study may benefit clinics that are implementing NBS for LSDs as the adoption of these practices preemptively may reduce the burden of that challenge.

1

**Summary**

- Multi‐tier screen protocols and collaboration with subspecialty providers were the most helpful changes adopted when lysosomal disorders were added to the state newborn screening panel.

## 2 Introduction

Lysosomal storage disorders (LSDs) are heritable inborn errors of metabolism that alter lysosomal enzyme functions and often result in a buildup of substrate [1]. LSDs were first introduced on the Recommended Universal Screening Panel (RUSP) in 2015 with the addition of Pompe disease and in 2016 with mucopolysaccharidosis type I (MPS I) [2], which has helped to save thousands of children's lives [3]. Prior to newborn screening (NBS) for LSDs, most patients were identified after symptom onset. Without early diagnosis and treatment, most LSDs will have progressed past the point of maximum therapeutic benefit. This leaves those affected with lasting sequelae and shortened lifespans [4].

NBS for LSDs typically employs a combination of enzymatic testing, assessment of biomarkers, and genetic testing [5, 6]. Some states utilize a single‐tier approach that only involves enzymatic testing, while others take on a multitier stepwise approach that utilizes multiple screening techniques to help reduce the yield of false positives [7, 8]. Without the use of multitier testing, NBS methods can identify patients with late‐onset LSDs, carriers, and those with pseudo‐deficiencies, which can make it difficult for healthcare providers to interpret the NBS results [8, 9, 10].

Our study explored the experiences of healthcare providers after the addition of LSDs to NBS. We identified actions and modifications to protocols made after the implementation of NBS for LSDs. These results can inform healthcare providers and institutions to improve patient care by efficiently and effectively implementing LSDs to NBS.

## 3 Methods

### 3.1 Participants

This study was deemed exempt by the Indiana University Institutional Review Board. Healthcare providers with experience working with LSDs within the last five years were eligible to participate in the research study. A link to the online survey was distributed via email to individuals at LSD clinics and two genetic counselors’ organizations at the end of June 2024. Data collection ended in October 2024. Data were collected and managed using REDCap electronic data capture tools hosted at Indiana University [11, 12]. Details are provided in Supporting Information.

### 3.2 Instrumentation

The survey questions included demographics (Table S1) and LSD‐related information (Table 1). Participants ranked the LSD they felt benefitted patients the most and the LSD benefitting patients the least. Participants could choose from any of 11 logistical challenges encountered before the addition of LSDs to NBS (e.g., adequate screening protocols, interpretation of follow‐up testing) and from any of 6 changes to clinical infrastructure (Table 2). Participants answered the same questions from the perspective of *after* the addition of LSDs to NBS. We also asked participants to select any/all of the same 6 actions that were *not* taken that they believed would have addressed challenges that arose with the addition of LSDs to NBS (Table S2).

**TABLE 1:** Summary information for lysosomal disorders.

LSDs on NBS	Number of participants (*N* = 45)	Percent (%)
MPS I	41	91
Pompe disease	41	91
MPS II	17	38
Krabbe disease	17	38
Fabry disease	10	22
Gaucher disease	9	20
Niemann‐Pick disease	5	11
Unsure	3	7
None of the above	2	4

Abnormal newborn screening	Median	Mean
Number of abnormal NBS in a year	30	75
Number of abnormal NBS for LSDs in a year	13	17

Abbreviations: LSD, lysosomal storage disorder; MPS, mucopolysaccharidosis; NBS, newborn screening.

**TABLE 2:** Summary information for challenges and changes made pre‐ and post‐addition of lysosomal disorders to newborn screening.

	Pre‐addition	Post‐addition
Logistical challenges	Percent (*N* = 32)	Percent (*N* = 28)
NBS results interpretation	75%	64%
Adequate screening protocols	63%	25%
Interpretation of follow‐up testing	56%	64%
Ordering of follow‐up testing	47%	32%
Insurance coverage of follow‐up testing	47%	25%
Timely treatment of patients	44%	11%
Patient compliance	41%	39%
Timely scheduling of patients	34%	25%
Access to treatment at your facility	22%	14%
Access to knowledgeable providers	19%	14%
Other	13%	11%

Were There Changes to Clinical Structure Prior to LSDs on NBS		
Yes	38%	N/A
No	62%	N/A

Changes to Clinical Structure		
Identified other subspeciality providers to collaborate on patient care	50%	68%
Provided additional training of existing staff	50%	50%
Held multidisciplinary team meetings	42%	43%
Made changes to clinic schedule	42%	39%
Recruited new medical providers	8%	14%
Hired new support staff	0%	11%
Other	33%	7%

Participants selected any/all of 10 actions they personally took (or were advised to take) to address challenges that they faced (e.g., hired additional staff and/or providers, collaborated with other healthcare providers), and to choose the actions that were the most and the least beneficial. Participants selected up to 6 resources they used to gather additional information involving LSDs and/or NBS (e.g., publications, institutional resources, and outside experts), and then ranked the most helpful resource. Participants could provide comments in open‐ended responses after ranking the LSD most benefitting patients, least benefitting patients, and the action that was most beneficial (Table S3). The full survey and data file are provided in Supporting Information.

### 3.3 Data Analysis

We performed a McNemar change test to compare if participants changed their top‐ranked LSD pre vs. post, for LSDs with at least *N* = 10 in the pre‐NSB. The significant threshold for reported results is alpha = 0.05. As this is an exploratory study, we did not correct for multiple testing. Means, standard deviation (SD), and standard error (SE) are provided as descriptive statistics.

## 4 Results

The survey reached approximately 5000 healthcare providers, including 48 LSD healthcare providers and 44 NBS coordinators emailed directly. 48 people started the survey, resulting in a response rate of ~1%. *N* = 32 completed at least half of the survey, and of those, 28 fully completed the survey. Results are reported for all 48 participants. The majority of participants were genetic counselors (65%), and almost all participants had experience working with abnormal NBS (93%). The most common LSDs on NBS in the participants’ states were MPS I (91%) and Pompe disease (91%), followed by 37% who selected Fabry and Krabbe. All results are described in Tables S1 and 1.

### 4.1 Anticipated Challenges and Preemptive Adjustments

The most common anticipated challenges were NBS results interpretation (75%), adequate screening protocols (62%), and interpretation of follow‐up testing (56%). 38% indicated changes to their clinical infrastructure in anticipation of the addition of LSDs. Most changes involved additional training of existing staff (50%) and the identification of other subspecialty providers to collaborate on patient care (50%). All results are provided in Table 2.

### 4.2 Experienced Challenges and Subsequent Adjustments

The most common challenges after the addition of LSDs to NBS were NBS results interpretation (64%) and interpretation of follow‐up testing (64%). The most common changes implemented in response to the addition of LSDs were identifying other subspecialty providers to collaborate on patient care (68%), providing additional training to existing staff (50%), and holding multidisciplinary meetings (43%). One of the most frequently selected actions that participants wished had been made was the hiring of new support staff (43%) or new medical providers (32%). Personal changes included collaborating with other healthcare providers (54%) and accessing sponsored genetic testing (54%). The most frequently selected were publications (100%), outside experts (82%), and conferences and seminars (71%). All summary results are provided in Table 2 and Table S2.

### 4.3 Utility of Specific LSDs on Newborn Screening

**FIGURE 1:**
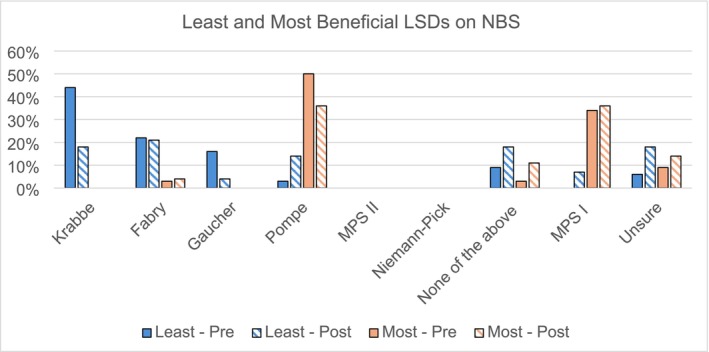
Least and most beneficial lysosomal disorders (LSDs) on newborn screening (NBS) from the perspective of prior and post addition of LSDs to NBS.

Participants were asked to rank the LSD that would benefit patients the least before the addition of LSDs to NBS (*N* = 32 responses total) and after being added to NBS (*N* = 28 responses total, Figure 1). Krabbe was the most common top‐ranked LSD benefiting patients the least pre‐addition (43.7%). However, 71.4% of those participants ranked a different disorder as benefiting patients the least post‐addition (McNemar *p* = 0.0067). There were insufficient numbers to test the remaining disorders for the pre‐addition rankings. Pompe and MPS I were the top‐ranked disorders most frequently chosen as benefiting patients the most, both pre‐addition (50.0% and 34.5%, respectively) and post‐addition (35.7% and 35.7%). Of those who ranked Pompe as the top disorder, 90% ranked it as the top disorder post‐addition (McNemar *p* = 1.0). Similarly, 90% of those who selected MPS I also ranked it as the top disorder post‐addition (McNemar *p* = 1.0). There were no significant changes between pre vs. post for any other disorders (all *p* > 0.08).

## 5 Discussion

This study revealed that the greatest challenges healthcare providers anticipated with the addition of LSDs to NBS were the interpretation of newborn screen results and having adequate screening protocols. This could be due to the use of single‐tier enzymatic testing, which is used in many states as an initial screen for LSDs on NBS [6]. Previous studies reported that a common outcome in single‐tier systems of NBS for LSDs is high false‐positive rates [9, 13] and that a multitier approach reduced uncertainty and false positives 5 [5], minimized ambiguity, and in some cases shortened the time it took to obtain a diagnosis [9]. Additionally, a multitier system can potentially help resolve common issues such as scheduling demands, determining and conveying the urgency of treatment, and limiting undue anxiety to families [10, 14].

This study indicates that after the addition of LSDs to NBS, many providers no longer believed the adequacy of screening protocols was a challenge, as only 25% selected it post‐addition compared to 63% that chose it prior to the addition. This decrease may reflect increased confidence in the sensitivity of initial NBS for LSDs [15]. However, a typical NBS protocol prioritizes the identification of all at‐risk babies with the understanding that there may be a high false‐positive rate [5, 16]. Despite the high sensitivity of NBS for LSDs, the majority of participants indicated that the interpretation of NBS results and follow‐up testing remains a challenge. The difficulty in interpretation may be due to the low specificity of single‐tier NBS, as enzyme analysis, the most common first‐ or single‐tier NBS assay, cannot differentiate between patients with late‐onset LSDs, carriers, or those with pseudo‐deficiencies [17]. See Table S3 for participant quotes supporting this finding. The implementation of multitier testing for LSDs can help increase sensitivity by incorporating molecular or biomarker analysis. This allows for the identification of at‐risk newborns, while simultaneously increasing specificity by clarifying patient phenotype [17, 18]. A multitier system also offers healthcare providers more insight into the expected prognosis of a patient and reduces difficulties associated with the interpretation of NBS results [5]. Longitudinal studies surveying LSD healthcare providers could allow identification of specific adjustments made, such as a multitier system.

Despite the various challenges associated with the inclusion of LSDs on NBS, existing infrastructure, such as sponsored genetic testing programs, can help mitigate some of the logistical complexities [19]. Approximately half of the participants reported that they accessed sponsored genetic testing for patients. This allows patients who screen positive for an LSD to have genetic testing at no out‐of‐pocket cost when it is not included as part of the NBS testing algorithm. Sponsored genetic testing ensures more equitable access to testing across all patients and provides the ability to have a more timely diagnosis [20, 21, 22]. This is particularly beneficial in the NBS process, where a quick turnaround time and access to care are essential. This system benefits both patients and healthcare clinics by alleviating the additional challenges associated with genetic testing, such as obtaining and navigating insurance coverage, a challenge that was selected by almost half the sample.

Collaboration was a key finding in these results. Identifying subspecialty care providers was the most common adaptation to the inclusion of LSDs in NBS, almost half of participants implemented multidisciplinary meetings, and just over half sought collaboration with other clinicians as a personal action taken. See Table S3 for participant quotes supporting multidisciplinary care. Such collaborations have been shown to improve patient outcomes [23] and management [24, 25] and align with guidelines for providing optimal care for patients with LSDs identified by NBS [26, 27, 28].

One LSD ranked by just under half of healthcare providers as being the least beneficial for patients was Fabry disease. Previous research demonstrated healthcare providers did not prioritize Fabry as a condition for NBS as it is not a neonatal/early childhood condition [29]. In fact, healthcare providers described late‐onset conditions such as Fabry disease, Gaucher disease, and certain forms of Pompe disease and MPS I as “time‐bombs” with parents waiting for their child to develop the disease [8, 29]. Early identification and treatment, one of the key tenets of NBS, would not apply to patients with Fabry disease as intervention in early childhood is not indicated by current medical literature. Identification of affected relatives is not one of the traditional goals of NBS as it does not benefit the patient directly. However, qualitative studies exploring the psychosocial impacts on patients identified as carriers by NBS or patients with late‐onset conditions could provide valuable insight into the value of testing from the patient perspective and how to appropriately counsel these patients. See Table S3 for participant quotes detailing their opinions on certain LSDs on NBS.

One of the strengths of this study is the broad range of expertise across the respondents. However, the number of participants was small, primarily due to the limited population of healthcare providers working with patients with LSDs. It is possible that the results may not be representative of all health providers working in the field of LSDs, due to the combination of a small sample size and incomplete survey responses. In addition, participants reported different LSDs on the NBS in their respective states, which may have influenced their selection of the most and least beneficial LSDs to have on NBS. This retrospective study asked participants to recall their thoughts and experiences prior to the addition of LSDs to NBS, which could have introduced a recall bias.

These results support the RUSP's addition of LSDs to NBS and the value of collaboration. These results could significantly benefit clinics in states where LSDs have not yet been added to NBS as they can consider adopting some of these practices preemptively, mitigating the impact of challenges on patient care.

## Author Contributions

**A. Terrell:** conceptualization, methodology, investigation, resources, data curation, writing – original draft, funding acquisition. **L. Wetherill:** methodology, formal analysis, data curation, writing – review and editing, supervision, project administration. **B. Graham:** writing – review and editing. **M. McPheron:** methodology, writing – review and editing. **K. Sapp:** conceptualization, methodology, Writing – review and editing, Supervision.

## Ethics Statement

This study was approved by the Institutional Review Board at Indiana University on 5/1/2024 (Protocol #23065).

## Consent

Consent was obtained from all study participants via consent statement before the start of the survey. All research was conducted in accordance with The Code of Ethics of the World Medical Association.

## Conflicts of Interest

The authors declare no conflicts of interest.

## Supporting information


Data S1.

Data S2.

Data S3.

## Data Availability

The data that supports the findings of this study are available in the supplementary material of this article.
