# Supplementary material for: Exploratory Study on the Challenges of Newborn Screening for Lysosomal Storage Disorders Emphasizes the Need for Multitier Testing and Collaborative Approaches to Management
Source: JIMD Rep. 2025 Jun 16;66(4):e70027. doi: 10.1002/jmd2.70027 (PMC12169912; doi:10.1002/jmd2.70027)
Supplement: Supplementary file 1 — Data S1. [file JMD2-66-e70027-s002.docx]

Supplemental Material

Methods:

This quantitative study was deemed exempt by the Indiana University Institutional Review Board. Healthcare providers with experience working with LSDs within the last five years were eligible to participate in the research study to ensure participants had relevant experience, as LSDs were not on NBS until 2015. A link to the online survey was distributed via two emails to the National Society of Genetic Counselors and the American Board of Genetic Counseling listservs at the end of June 2024. A reminder email was sent approximately two weeks after the initial emails. Emails with a link were also sent directly to healthcare providers employed by lysosomal storage disorder clinics on June 10^th^, 2024. A reminder email was sent approximately a month after the initial email to the LSD clinic providers. All participants who completed the survey had the option to provide their email in a separate REDCap survey to be eligible to receive one of five $50 Amazon gift cards in a random drawing. Data collection ended October 2024.

Supplemental Table 1: Demographics

| **Current Position (N = 48)** | **Number of Participants** | **Percent** |
| --- | --- | --- |
| Genetic Counselor | 31 | 65% |
| Medical Doctor | 9 | 19% |
| Registered Nurse | 2 | 4% |
| Nurse Practitioner | 1 | 2% |
| Support Staff | 1 | 2% |
| Physician Assistant | 0 | 0% |
| Other | 4 | 8% |
| **Total Years Spent in Position**  **(N = 48)** |  |  |
| 0-5 years | 25 | 52% |
| 5-10 years | 15 | 31% |
| 10+ years | 8 | 17% |
| **Experience Working with Abnormal NBS (N = 45)** |  |  |
| Yes | 42 | 93% |
| No | 3 | 7% |

NBS = newborn screening

Supplemental Table 2: Summary information for actions taken and resources utilized post-addition of lysosomal disorders to newborn screening.

|  | **Post-Addition** |
| --- | --- |
| **Actions that They Wish Had Been Done** | **(N = 28)** |
| Hiring of new support staff | 43% |
| Recruitment of new medical providers | 32% |
| Additional training of existing staff | 14% |
| Held multidisciplinary team meetings | 11% |
| Changes in clinic schedule | 7% |
| Collaboration with other subspeciality providers | 0% |
| Other | 39% |
| **Personal Actions** | **N = 28** |
| Collaborated with other healthcare providers | 54% |
| Accessed sponsored genetic testing | 54% |
| Improved care coordination process | 50% |
| Identified appropriate resources for patients | 46% |
| Offered more telehealth appointments | 43% |
| Established management protocol | 43% |
| Provided education across healthcare system | 29% |
| Implemented scheduling changes | 17% |
| Hired additional staff and/or providers | 7% |
| Altered prior authorization process | 4% |
| Other | 18% |
| **Resources** | **N = 28** |
| Publications | 100% |
| Outside experts | 82% |
| Conferences/Seminars | 71% |
| Colleagues at your institution | 54% |
| Google | 29% |
| Institutional resources | 11% |
| Other | 7% |

Supplemental Table 3. Open-ended responses from participants. Participants were offered to elaborate on the following: 1) Why they believe the top-ranked lysosomal disorder would benefit patients the least (by being added to the newborn screening), 2) Why they believe the top-ranked lysosomal disorder would benefit patients the most (by being added to the newborn screening), and 3) why a multidisciplinary care approach made by a participant’s institution was chosen as being most beneficial.

| **1) Why the top-ranked lysosomal disorder would benefited patients the least** |
| --- |
| Numerous false positives were generated. PCPs and other providers occasionally provided alarming news to families under false pretense. |
| We do not start treatment until an individual is symptomatic so the early identification at newborn stage (when they most likely don't have symptoms) is not entirely helpful |
| Severity makes treatment of uncertain benefit |
| We have only had one Krabbe referral and that child was transplanted at Duke the same month of identification, so the family thinks of it as a success. We have only had one MPS1 referral that was positive as well. We have many late onset pompe that we think is benefitting patients but the families don't always agree. |
| We do not start treatment until an individual is symptomatic so the early identification at newborn stage (when they most likely don't have symptoms) is not entirely helpful |
| It's a small population. Not everyone supports the idea of transplant being beneficial based on outcomes and QOL. |
| We don't have 2nd tier testing, so 99.9% of the screens are not actually Krabbe disease; causes families undo stress. Additionally, we only had 1 baby test positive in 7 years and baby passed away during transplant process. I don't consider the treatment very good. |
| Is a stem cell transplant by 1 month really equitable treatment for all? Do we know outcomes are improved for babies who get a transplant? Do ALL families want this information (ex like on an opt out program like newborn screening), or just the families who have good access to care, good insurance coverage, etc |
| There is no immediate symptomatology in the newborn period, and more specific guidelines for when to start asymptomatic patients are needed. This creates a large pool of patients in waiting. We have also struggled with the A143T variant being so prevalent with conflicting interpretations of its pathogenicity. There is benefit to cascade screening for family members, but this is not traditionally a goal of newborn screening. |
| Our state screens for MPS I and Pompe disease, but I don't think that one has been more or less beneficial than the other. |
| Very few positive cases have required treatment |
| Haven't had any true diagnoses |
| I don't feel I've had sufficient experience to make this call just yet regarding true diagnoses, but I chose MPS1 only because the overwhelming majority of positive screens have been pseudodeficiency and thus has created more challenges than benefits so far. |
| Majority of Gaucher patients in our state have Type I, which I feel is not as critical for early intervention like the LSDs that are on the RUSP. |
| Majority of patients have late-onset Fabry disease and may not require treatment for many years. |
| Using ERT in the infantile form has very limited success (unless applied in the prenatal period which doesn't apply here). It's impossible to predict in most cases if baby will develop juvenile or late onset form of the disease - saying to a family that symptoms could begin at 2 years up to 60+ years feel cruel, that degree of uncertainty and anxiety and increased medicalization - especially in the setting of a VUS. |
| diagnosing children with late onset disorders of uncertain onset - significant uncertainty |
| No treatment |
| Have not identified any confirmed IOPD thus far, but many "suspected" LOPD with uncertain results |
| For Fabry- we identify so many VUSs or A143T that we then are tasked to follow over time/screen other family members. I think we end up overmedicalizing a lot of individuals who do not need low term follow up. For Krabbe, our NBS assay has changed a lot over the past several years- we end up identifying a lot of individuals with just carrier status, who are initially told by their PCP that they have this devastating disorder (because of course the PCP is not interpreting the result correctly)- I think it NBS for EIKD is very important, but I do not think it is appropriate to fearmonger these carrier-only families and we should not be screening for carrier status on NBS. |
| It is not a neonatal or even childhood disease |
|  |
| **2) Why the top-ranked lysosomal disorder would benefited patients the most** |
| The screening protocol seemed highly precise and sensitive for MPS I. Thus, we're not aware of issues with false positives |
| Early initiation of ERT in patients with IOPD have led to early resolution/improvement of cardiac symptoms |
| Treatment of IOPD is lifesaving |
| Early detection and monitoring for many that may or will develop disease in the future |
| We have clear management guidelines for MPSI |
| The IOPD kids are alive - but we are finding they still have other complications now that they are surviving. |
| For infantile onset Pompe, early treatment truly changes the outcome. Eliminates the diagnostic odyssey. |
| The echo recommendation asap could catch any possibly fatal cardiomyopathies |
| Timely identification allows patients to undergo HSCT within the recommended 3 month timeframe and receive the maximum benefit of this intervention. The challenge is in identifying which patients have severe vs attenuated disease. |
| Early diagnosis and treatment clearly associated with better outcome |
| Much more common than originally anticipated and now can follow over time to initiate treatment when appropriate. Also have identified MANY affected mothers who were previously undiagnosed and now benefit from treatment. |
| I chose Pompe disease because I have the most experience with this, and because I have experienced at least one patient make drastic improvements after beginning early treatment with enzyme replacement therapy. |
| I believe Pompe disease has benefitted the most because it decreases the time to diagnosis for infantile onset patients. Prior to NBS, most of our patients were picked up at >5months of age, which significantly impacts outcome in a negative way. |
| Both Hurler and Scheie patients benefit from early diagnosis and monitoring for treatment purposes, but also awareness of other health issues or surgical risk factors (ie. increased risk of complications during intubation). Since many patients require surgeries (ie. umbilical hernia, tonsil and adenoidectomy, ear tubes, etc.) this can be pertinent even if a patient isn't recommended HSCT or ERT. |
| BMT is actually very effective immediately after diagnosis although still has its limitations and risks |
| early diagnosis and treatment |
| No confirmed diagnoses of other LSDs yet |
| Access to HSCT if indicated. |
| Overall there is reasonable guidance to therapy |
|  |
| **3) Why a multidisciplinary care approach made by a participant’s institution was chosen as being most beneficial** |
| It facilitated exchange of knowledge which may be helpful in navigating uncertain results |
| The more folks who are involved and aware of NBS, the more supported patients can feel |
| Results and interpretation can be complicated. Having a team specialized in interpreting results and coordinating testing is beneficial for patients and their families. |
| There are so many unusual cases that come through NBS for LSDs that it is absolutely necessary to meet as a team to review these unusual or complex cases. |
