# Supplementary material for: Exploratory Study on the Challenges of Newborn Screening for Lysosomal Storage Disorders Emphasizes the Need for Multitier Testing and Collaborative Approaches to Management
Source: JIMD Rep. 2025 Jun 16;66(4):e70027. doi: 10.1002/jmd2.70027 (PMC12169912; doi:10.1002/jmd2.70027)
Supplement: Supplementary file 2 — Data S2. [file JMD2-66-e70027-s003.pdf]

Data Dictionary Codebook

Professional Development Project - Terrell (PID: 36089)

04/24/2024 12:34pm

Instruments

	#	Variable / Field Name	Field Label<br>*Field Note*	Field Attributes (Field Type, Validation, Choices, Calculations, etc.)
**Instrument: Addition Of Lysosomal Storage Disorders to Newborn Screening Survey - Terrell** (addition_of_lysosomal_storage_disorders_to_newborn) Enabled as survey				
	1	[participant_id]	Participant ID	text
	2	[current_occupation]	Section Header: *Demographics*<br>Current Position	dropdown, Required<br>1 Medical doctor<br>2 Genetic counselor<br>3 Registered nurse<br>7 Nurse practitioner<br>8 Physician assistant<br>4 Support staff<br>5 Management<br>6 Other<br><br>Question number: 1
	3	[current_occupation_other]<br><br>Show the field ONLY if: [current_occupation] = '6'	Other - Please write the title of your current position if not listed.	notes, Required
	4	[current_occupation_years]	How many years have you been working in your current position? If less than 1 year, please enter 1.	text (integer, Min: 1, Max: 70), Required
	5	[lsds_experience]	Do you have experience working with LSD patients?	yesno, Required<br>1 Yes<br>0 No<br><br>Stop actions on 0
	6	[curr_prev_occupation_lsd]<br><br>Show the field ONLY if: [lsds_experience] = '1'	Is this experience from your current position or a previous position?	radio, Required<br>1 Current Position<br>2 Previous Position
	7	[previous_occupation_title]<br><br>Show the field ONLY if: [curr_prev_occupation_lsd] = '2'	Please select the title of your previous position.	dropdown, Required<br>1 Medical doctor<br>2 Genetic counselor<br>3 Registered nurse<br>7 Nurse practitioner<br>8 Physician assistant

	#	Variable / Field Name	Field Label	Field Attributes
				4 Support staff<br>5 Management<br>6 Other
	8	[previous_occupation_years]<br><br>Show the field ONLY if:<br>[curr_prev_occupation_lsd] = '2'	How many years were you in that role? If less than 1 year, please enter 1.	text (integer, Min: 1, Max: 70), Required
	9	[previous_occupation_years_left]<br><br>Show the field ONLY if:<br>[curr_prev_occupation_lsd] = '2'	How many years ago did you leave that role?	dropdown, Required<br>1 1<br>2 2<br>3 3<br>4 4<br>5 5<br>6 6+<br><br>Stop actions on 6
	10	[abnormal_nbs_experience]	Do you have experience taking care of patients with abnormal NBS?	yesno, Required<br>1 Yes<br>0 No
	11	[curr_prev_occupation_nbs]	Is your experience with patients with abnormal NBS from your current position or a previous position?	radio, Required<br>1 Current Position<br>2 Previous Position
	12	[curr_prev_occu_years_nbs]<br><br>Show the field ONLY if:<br>[curr_prev_occupation_nbs] = '2'	How many years were you in that role? If less than 1 year, please enter 1.	text (integer, Min: 1, Max: 70), Required
	13	[curr_prev_occu_left_nbs]<br><br>Show the field ONLY if:<br>[curr_prev_occupation_nbs] = '2'	How many years ago did you leave that role? If less than 1 year, please enter 1.	text (integer, Min: 1, Max: 70), Required
	14	[lsd_nbs_state]	What LSDs are on NBS in your state? Please select all that apply.	checkbox, Required<br>1 lsd_nbs_state___1 Mucopolysaccharidosis type I (MPS I)<br>2 lsd_nbs_state___2 Mucopolysaccharidosis type II (MPS II)<br>3 lsd_nbs_state___3 Fabry disease<br>4 lsd_nbs_state___4 Pompe disease<br>5 lsd_nbs_state___5 Krabbe disease<br>6 lsd_nbs_state___6 Gaucher disease<br>7 lsd_nbs_state___7 Niemann-Pick disease<br>8 lsd_nbs_state___8 None of the above

				9 | lsd_nbs_state___9 | Unsure
	15	[nbs_results]	How many abnormal NBS results do you see in one year? Please estimate.	slider (number, Min: 0, Max: 1000), Required<br>Slider labels: 1, , 1000<br>Custom alignment: RH
	16	[frequency_lsd]	How frequently do you see patients with abnormal newborn screening results for LSDs in a year? Please estimate.	slider (number, Min: 0, Max: 100), Required<br>Slider labels: 1, 50, 100<br>Custom alignment: RH
	17	[pre_logistic_challenges]	Section Header: *Pre-Addition of Lysosomal Storage Disorders to Newborn Screening: (LSDs = Lysosomal Storage Disorders, NBS = Newborn Screening) Please answer the following questions with what you believed before the addition of LSDs to NBS.*<br>Before the addition of LSDs to NBS, what logistical challenges did you expect to encounter? Please select all that apply.	checkbox, Required<br>1 | pre_logistic_challenges___1 | Adequate screening protocols<br>2 | pre_logistic_challenges___2 | NBS results interpretati<br>3 | pre_logistic_challenges___3 | Interpretati of follow-u testing<br>4 | pre_logistic_challenges___4 | Ordering o follow-up testing<br>5 | pre_logistic_challenges___5 | Insurance coverage o follow-up testing<br>6 | pre_logistic_challenges___6 | Timely scheduling patients<br>7 | pre_logistic_challenges___7 | Timely treatment patients<br>8 | pre_logistic_challenges___8 | Access to knowledge providers<br>9 | pre_logistic_challenges___9 | Access to treatment your facility<br>10 | pre_logistic_challenges___10 | Access to treatment from patien insurance<br>11 | pre_logistic_challenges___11 | Patient compliance<br>12 | pre_logistic_challenges___12 | Other
	18	[pre_logistic_chall_other]<br>Show the field ONLY if:<br>[pre_logistic_challenges(12)] = '1'	Please specify.	notes
	19	[before_the_addition_of_lsd]	Before the addition of LSDs to NBS, how difficult did you perceive the following	descriptive

			psychosocial challenges to be?	
	20	[pre_deliver_results]	Delivering results to unsuspecting families	radio (Matrix), Required<br>1 Not difficult<br>2 Somewhat difficult<br>3 Very difficult
	21	[pre_explan_follow_up]	Explaining and following up on uncertain results	radio (Matrix), Required<br>1 Not difficult<br>2 Somewhat difficult<br>3 Very difficult
	22	[pre_emphasiz_urgency]	Emphasizing the urgency of treatment	radio (Matrix), Required<br>1 Not difficult<br>2 Somewhat difficult<br>3 Very difficult
	23	[pre_identif_barriers]	Identifying barriers to compliance/long-term follow-up	radio (Matrix), Required<br>1 Not difficult<br>2 Somewhat difficult<br>3 Very difficult
	24	[pre_psych_other]	Other - Please explain	radio (Matrix)<br>1 Not difficult<br>2 Somewhat difficult<br>3 Very difficult
	25	[pre_psych_oth]<br>Show the field ONLY if: [pre_psych_other] = '1' or [pre_psych_other] = '2' or [pre_psych_other] = '3'	If other, please explain and rate difficulty.	notes, Required<br>Custom alignment: RH
	26	[pre_clinic_implement]	In anticipation of the addition of LSDs to NBS, did your clinic implement any changes to clinical infrastructure?	yesno, Required<br>1 Yes<br>0 No<br>Custom alignment: RH
	27	[pre_what_changes_impl]<br>Show the field ONLY if: [pre_clinic_implement] = '1'	What changes were implemented? Please select all that apply.	checkbox, Required<br>1 pre_what_changes_impl___1 Hired new support staf<br>2 pre_what_changes_impl___2 Provided additional training of existing staff<br>3 pre_what_changes_impl___3 Held multidiscipli team meeti<br>4 pre_what_changes_impl___4 Made chang to clinic

				schedule<br>5 pre_what_changes_impl___5 Recruited additional medical providers<br>6 pre_what_changes_impl___6 Identified ot[illegible] subspeciality providers to collaborate [illegible] patient care<br>7 pre_what_changes_impl___7 Other
	28	[pre_clin_other]<br>Show the field ONLY if:<br>[pre_what_changes_impl(7)] = '1'	Please specify.	notes, Required<br>Custom alignment: RH
	29	[pre_least_ben]	What LSDs did you believe would benefit patients the least when added to NBS? Please select all that apply.	checkbox, Required<br>1 pre_least_ben___1 Mucopolysaccharidos[illegible] type I (MPS I)<br>2 pre_least_ben___2 Mucopolysaccharidos[illegible] type II (MPS II)<br>3 pre_least_ben___3 Fabry disease<br>4 pre_least_ben___4 Pompe disease<br>5 pre_least_ben___5 Krabbe disease<br>6 pre_least_ben___6 Gaucher disease<br>7 pre_least_ben___7 Niemann-Pick disease<br>8 pre_least_ben___8 None of the above<br>9 pre_least_ben___9 Unsure
	30	[pre_least_ben_rank]	Of the LSDs that you selected in the previous question, which did you expect would benefit patients the least?	dropdown, Required<br>1 Mucopolysaccharidosis type I (MPS I)<br>2 Mucopolysaccharidosis type II (MPS II)<br>3 Fabry disease<br>4 Pompe disease<br>5 Krabbe disease<br>6 Gaucher disease<br>7 Niemann-Pick disease<br>8 None of the above<br>9 Unsure
	31	[pre_most_ben]	What LSDs did you believe would benefit patients the most when added to NBS? Please select all that apply.	checkbox, Required<br>1 pre_most_ben___1 Mucopolysaccharidosi[illegible] type I (MPS I)<br>2 pre_most_ben___2 Mucopolysaccharidosi[illegible] type II (MPS II)<br>3 pre_most_ben___3 Fabry disease<br>4 pre_most_ben___4 Pompe disease

			5 pre_most_ben___5 Krabbe disease<br>6 pre_most_ben___6 Gaucher disease<br>7 pre_most_ben___7 Niemann-Pick disease<br>8 pre_most_ben___8 None of the above<br>9 pre_most_ben___9 Unsure
32	[pre_most_ben_rank]	Of the LSDs that you selected in the previous question, which did you expect would benefit patients the most?	dropdown, Required<br>1 Mucopolysaccharidosis type I (MPS I)<br>2 Mucopolysaccharidosis type II (MPS II)<br>3 Fabry disease<br>4 Pompe disease<br>5 Krabbe disease<br>6 Gaucher disease<br>7 Niemann-Pick disease<br>8 None of the above<br>9 Unsure
33	[before_the_addition_of]	Before the addition of LSDs to NBS, what was your level of confidence in each of the following areas?	descriptive
34	[pre_natural_history]	Natural history of the condition	radio (Matrix), Required<br>1 Not confident<br>2 A little confident<br>3 Neutral<br>4 Moderately confident<br>5 Very confident
35	[pre_interpretation]	Interpretation of genetic testing results	radio (Matrix), Required<br>1 Not confident<br>2 A little confident<br>3 Neutral<br>4 Moderately confident<br>5 Very confident
36	[pre_disease_treatment]	Disease-specific treatment (ERT, SRT, BMT, etc.)	radio (Matrix), Required<br>1 Not confident<br>2 A little confident<br>3 Neutral<br>4 Moderately confident<br>5 Very confident
37	[pre_implementation]	Implementation of management guidelines	radio (Matrix), Required<br>1 Not confident<br>2 A little confident<br>3 Neutral

				4 Moderately confident<br>5 Very confident
	38	[pre_manag_unexpected]	Management of unexpected disease complications	radio (Matrix), Required<br>1 Not confident<br>2 A little confident<br>3 Neutral<br>4 Moderately confident<br>5 Very confident
	39	[pre_patient_resources]	Patient support resources	radio (Matrix), Required<br>1 Not confident<br>2 A little confident<br>3 Neutral<br>4 Moderately confident<br>5 Very confident
	40	[post_log_challenges]	Section Header: *Post-Addition of Lysosomal Storage Disorders to Newborn Screening: (LSDs = Lysosomal Storage Disorders, NBS = Newborn Screening) Please answer the following questions with what you believed after the addition of LSDs to NBS.*<br>After the addition of LSDs to NBS, what logistical challenges did you face? Select all that apply.	checkbox, Required<br>1 post_log_challenges___1 Adequate screening protocols<br>2 post_log_challenges___2 NBS results interpretation<br>3 post_log_challenges___3 Interpretation of follow-up testing<br>4 post_log_challenges___4 Ordering of follow-up testing<br>5 post_log_challenges___5 Insurance coverage of follow-up testing<br>6 post_log_challenges___6 Timely scheduling of patients<br>7 post_log_challenges___7 Timely treatment of patients<br>8 post_log_challenges___8 Access to knowledgeabl providers<br>9 post_log_challenges___9 Access to treatment at your facility<br>10 post_log_challenges___10 Patient compliance<br>11 post_log_challenges___11 Other

	41	[post_log_challenge_oth]<br><br>Show the field ONLY if:<br>[post_log_challenges(11)] = '1'	Please specify.	notes, Required<br>Custom alignment: RH
	42	[how_difficult_were_the_fol]	How difficult were the following psychosocial challenges after the addition of LSDs to NBS?	descriptive
	43	[post_deliver_results]	Delivering results to unsuspecting families	radio (Matrix), Required<br>1 Not difficult<br>2 Somewhat difficult<br>3 Very difficult
	44	[post_explan_follow_up]	Explaining and following up on uncertain results	radio (Matrix), Required<br>1 Not difficult<br>2 Somewhat difficult<br>3 Very difficult
	45	[post_emph_urgency]	Emphasizing the urgency of treatment	radio (Matrix), Required<br>1 Not difficult<br>2 Somewhat difficult<br>3 Very difficult
	46	[post_identify_barriers]	Identifying barriers to compliance/long-term follow-up	radio (Matrix), Required<br>1 Not difficult<br>2 Somewhat difficult<br>3 Very difficult
	47	[post_psycho_other]	Other - Please explain	radio (Matrix)<br>1 Not difficult<br>2 Somewhat difficult<br>3 Very difficult
	48	[post_psycho_oth]<br><br>Show the field ONLY if:<br>[post_psycho_other] = '1' or [post_psycho_other] = '2' or [post_psycho_other] = '3'	If other, please explain and rate difficulty.	notes, Required
	49	[post_lsd_least_ben]	What LSDs do you believe have benefited patients the least by being added to NBS? Please select all that apply.	checkbox, Required<br>1 post_lsd_least_ben___1 Mucopolysacchar type I (MPS I)<br>2 post_lsd_least_ben___2 Mucopolysacchar type II (MPS II)<br>3 post_lsd_least_ben___3 Fabry disease<br>4 post_lsd_least_ben___4 Pompe disease<br>5 post_lsd_least_ben___5 Krabbe disease<br>6 post_lsd_least_ben___6 Gaucher disease<br>7 post_lsd_least_ben___7 Niemann-Pick dis

	#	Variable	Field Label	Field Attributes
				8 post_lsd_least_ben___8 None of the abov<br>9 post_lsd_least_ben___9 Unsure
	50	[post_lsd_ben_least_rank]	Of the LSDs that you selected in the previous question, which do you believe benefits patients the least?	dropdown, Required<br>1 Mucopolysaccharidosis type I (MPS I)<br>2 Mucopolysaccharidosis type II (MPS II)<br>3 Fabry disease<br>4 Pompe disease<br>5 Krabbe disease<br>6 Gaucher disease<br>7 Niemann-Pick disease<br>8 None of the above<br>9 Unsure
	51	[post_least_ben_elabor]<br>Show the field ONLY if: [post_lsd_least_ben(9)] = '1' or [post_lsd_least_ben(8)] = '1' or [post_lsd_least_ben(7)] = '1' or [post_lsd_least_ben(6)] = '1' or [post_lsd_least_ben(5)] = '1' or [post_lsd_least_ben(4)] = '1' or [post_lsd_least_ben(3)] = '1' or [post_lsd_least_ben(2)] = '1' or [post_lsd_least_ben(1)] = '1'	Please elaborate on why you believe this LSD has benefited patients the least.	notes<br>Custom alignment: RH
	52	[post_lsd_most_bene]	What LSDs do you believe have benefited patients the most by being added to NBS? Please select all that apply.	checkbox, Required<br>1 post_lsd_most_bene___1 Mucopolysaccha type I (MPS I)<br>2 post_lsd_most_bene___2 Mucopolysaccha type II (MPS II)<br>3 post_lsd_most_bene___3 Fabry disease<br>4 post_lsd_most_bene___4 Pompe disease<br>5 post_lsd_most_bene___5 Krabbe disease<br>6 post_lsd_most_bene___6 Gaucher disease<br>7 post_lsd_most_bene___7 Niemann-Pick d<br>8 post_lsd_most_bene___8 None of the abo<br>9 post_lsd_most_bene___9 Unsure
	53	[post_ben_most_rank]	Of the LSDs that you selected in the previous question, which do you believe benefits patients the most?	dropdown, Required<br>1 Mucopolysaccharidosis type I (MPS I)<br>2 Mucopolysaccharidosis type II (MPS II)<br>3 Fabry disease<br>4 Pompe disease<br>5 Krabbe disease

			6 Gaucher disease<br>7 Niemann-Pick disease<br>8 None of the above<br>9 Unsure
54	[post_ben_most_elabor]<br><br>Show the field ONLY if: [post_ben_most_rank] = '1' or [post_ben_most_rank] = '2' or [post_ben_most_rank] = '3' or [post_ben_most_rank] = '4' or [post_ben_most_rank] = '5' or [post_ben_most_rank] = '6' or [post_ben_most_rank] = '7' or [post_ben_most_rank] = '8' or [post_ben_most_rank] = '9'	Please elaborate on why you believe this LSD has benefited patients the most.	notes<br>Custom alignment: RH
55	[post_clinic_actions]	What specific actions has your work institution made to address some of the challenges that arose with the addition of LSDs to NBS? Please select all that apply.	checkbox, Required<br>1 post_clinic_actions___1 Hired new support staff<br>2 post_clinic_actions___2 Provided additional training of existing staff<br>3 post_clinic_actions___3 Held multidisciplinary team meetings<br>4 post_clinic_actions___4 Made changes to clinic schedule<br>5 post_clinic_actions___5 Recruited new medical providers<br>6 post_clinic_actions___6 Identified other subspeciality providers to collaborate on patient care<br>7 post_clinic_actions___7 Other
56	[post_clinic_actions_other]<br><br>Show the field ONLY if: [post_clinic_actions(7)] = '1'	Please specify.	notes, Required<br>Custom alignment: RH
57	[post_action_rank_least]	Of the actions you selected in the question above, which one do you believe has been the least beneficial?	dropdown, Required<br>1 Hired new support staff<br>2 Provided additional training of existing staff

				3 Held multidisciplinary team meetings<br>4 Made changes to clinic schedule<br>5 Recruited new medical providers<br>6 Identified other subspeciality providers to collaborate on patient care<br>7 Other
	58	[post_clinic_act_rank_elab_least]<br>Show the field ONLY if: [post_action_rank_least] = '1' or [post_action_rank_least] = '2' or [post_action_rank_least] = '3' or [post_action_rank_least] = '4' or [post_action_rank_least] = '5' or [post_action_rank_least] = '6'	Please elaborate on why these actions were the least helpful.	notes
	59	[post_action_rank_most]	Of the actions you selected in the question above, which one do you believe has been the most beneficial?	dropdown, Required<br>1 Hired new support staff<br>2 Provided additional training of existing staff<br>3 Held multidisciplinary team meetings<br>4 Made changes to clinic schedule<br>5 Recruited new medical providers<br>6 Identified other subspeciality providers to collaborate on patient care<br>7 Other
	60	[post_clinic_act_elab_most]<br>Show the field ONLY if: [post_action_rank_most] = '7' or [post_action_rank_most] = '6' or [post_action_rank_most] = '5' or [post_action_rank_most] = '4' or [post_action_rank_most] = '3' or [post_action_rank_most] = '2' or [post_action_rank_most] = '1'	Please elaborate on why these actions were the most helpful.	notes
	61	[actions_wish]	What specific actions were not taken that you believe would have addressed some of the challenges that arose with the addition of LSDs to NBS? Please select all that apply.	checkbox, Required<br>1 actions_wish___1 Hiring of new support staff<br>2 actions_wish___2 Additional training of existing staff<br>3 actions_wish___3 Held multidisciplinary

				team meetings<br>4 actions_wish___4 Changes in clinic schedule<br>5 actions_wish___5 Recruitment of new medical providers<br>6 actions_wish___6 Collaboration with of other subspeciality providers<br>7 actions_wish___7 Other
	62	[actions_wish_other]<br>Show the field ONLY if:<br>[actions_wish(7)] = '1'	Please specify.	notes, Required<br>Custom alignment: RH
	63	[actions_wish_why]<br>Show the field ONLY if:<br>[actions_wish(1)] = '1' and [actions_wish(2)] = '1' and [actions_wish(3)] = '1' and [actions_wish(4)] = '1' and [actions_wish(5)] = '1' and [actions_wish(6)] = '1' and [actions_wish(7)] = '1'	Please explain why you believe these actions would have addressed some of the challenges that arose with the addition of LSDs to NBS.	notes<br>Custom alignment: RH
	64	[post_provider_action]	What specific actions did you take (or were you advised to take) to address some of the challenges that you faced with the addition of LSDs to NBS? Please select all that apply.	checkbox, Required<br>1 post_provider_action___1 Implemented scheduling changes<br>2 post_provider_action___2 Hired additional staff and/or providers<br>3 post_provider_action___3 Offered more telehealth appointment<br>4 post_provider_action___4 Identified appropriate resources for patients<br>5 post_provider_action___5 Collaborated with other healthcare providers<br>6 post_provider_action___6 Altered prior authorization process<br>7 post_provider_action___7 Accessed sponsored genetic testing<br>8 post_provider_action___8 Improved care

				|	| coordination processes | | 9 | post_provider_action___9 | Provided education across healthcare system | | 10 | post_provider_action___10 | Established management protocol | | 11 | post_provider_action___11 | Other |
	65	[post_provider_action_oth]<br>Show the field ONLY if: [post_provider_action(11)] = '1'	Please specify.	notes, Required
	66	[post_action_person_least_rank]	Of the actions you selected in the question above, which were the least beneficial?	dropdown, Required<br>1 Implemented scheduling changes<br>2 Hired additional staff and/or providers<br>3 Offered more telehealth appointments<br>4 Identified appropriate resources for patients<br>5 Collaborated with other healthcare providers<br>6 Altered prior authorization process<br>7 Accessed sponsored genetic testing<br>8 Improved care coordination processes<br>9 Provided education across healthcare system<br>10 Established management protocol<br>11 Other
	67	[post_action_person_least_elab]<br>Show the field ONLY if: [post_action_person_least_rank] = '1' or [post_action_person_least_rank] = '2' or [post_action_person_least_rank] = '3' or [post_action_person_least_rank] = '4' or [post_action_person_least_rank] = '5' or [post_action_person_least_rank] = '6' or [post_action_person_least_rank] = '7' or [post_action_person_least_rank]	Please elaborate on why this action was the least helpful.	notes

		= '8' or [post_action_person_least_rank] = '9' or [post_action_person_least_rank] = '10' or [post_action_person_least_rank] = '11'		
	68	[post_action_person_most_rank]	Of the actions you selected in the question above, which were the most beneficial?	dropdown, Required<br>1 Scheduling changes<br>2 Hired additional staff and/or providers<br>3 Offered more telehealth appointments<br>4 Identified appropriate resources for patients<br>5 Collaborated with other healthcare providers<br>6 Altered prior authorization process<br>7 Accessed sponsored genetic testing<br>8 Improved care coordination processes<br>9 Provide education across healthcare system<br>10 Establish management protocol<br>11 Other
	69	[post_action_person_most_elab]<br>Show the field ONLY if: [post_action_person_most_rank] = '11' or [post_action_person_most_rank] = '10' or [post_action_person_most_rank] = '9' or [post_action_person_most_rank] = '8' or [post_action_person_most_rank] = '7' or [post_action_person_most_rank] = '6' or [post_action_person_most_rank] = '5' or [post_action_person_most_rank] = '4' or [post_action_person_most_rank] = '3' or [post_action_person_most_rank] = '2' or [post_action_person_most_rank] = '1'	Please elaborate on why this action was the most helpful.	notes
	70	[after_the_addition_of_lsds]	After the addition of LSDs to NBS, what is your level of confidence in each of the following areas?	descriptive
	71	[post_nat_history]	Natural history of the condition	radio (Matrix), Required<br>1 Not confident

				2 A little confident<br>3 Neutral<br>4 Moderately confident<br>5 Very confident
	72	[post_inter_of_genetic]	Interpretation of genetic testing results	radio (Matrix), Required<br>1 Not confident<br>2 A little confident<br>3 Neutral<br>4 Moderately confident<br>5 Very confident
	73	[post_disease_treatment]	Disease-specific treatment (ERT, SRT, BMT, etc.)	radio (Matrix), Required<br>1 Not confident<br>2 A little confident<br>3 Neutral<br>4 Moderately confident<br>5 Very confident
	74	[post_implement]	Implementation of management guidelines	radio (Matrix), Required<br>1 Not confident<br>2 A little confident<br>3 Neutral<br>4 Moderately confident<br>5 Very confident
	75	[post_manag_unexpected]	Management of unexpected disease complications	radio (Matrix), Required<br>1 Not confident<br>2 A little confident<br>3 Neutral<br>4 Moderately confident<br>5 Very confident
	76	[post_patient_resources]	Patient support resources	radio (Matrix), Required<br>1 Not confident<br>2 A little confident<br>3 Neutral<br>4 Moderately confident<br>5 Very confident
	77	[resources_lsd_nbs]	What resources did you use when seeking out additional information involving LSDs and/or NBS?	checkbox<br>1 resources_lsd_nbs___1 Publications<br>2 resources_lsd_nbs___2 Institutional resou<br>3 resources_lsd_nbs___3 Colleagues at you institution<br>4 resources_lsd_nbs___4 Outside experts

				5 resources_lsd_nbs___5 Google<br>6 resources_lsd_nbs___6 Conferences/Semi<br>7 resources_lsd_nbs___7 Other<br><br>Custom alignment: LV
	78	[resour_lsds_nbs_oth]<br><br>Show the field ONLY if: [resources_lsd_nbs(7)] = '1'	Please share or state said resource.	notes<br>Custom alignment: LV
	79	[resource_rank]	Of the resources you selected in the question above, please select the resource that was the most helpful.	dropdown, Required<br>1 Publications<br>2 Institutional resources<br>3 Colleagues at your institution<br>4 Outside experts<br>5 Google<br>6 Conferences/Seminars<br>7 Other
	80	[nbs_exp_help]<br><br>Show the field ONLY if: [abnormal_nbs_experience] = '1'	Did your prior experience with NBS help in the implementation of storage disorders on newborn screening?	radio (Matrix), Required<br>1 Not helpful<br>2 A little helpful<br>3 Moderately helpful<br>4 Very helpful
	81	[nbs_no_exp_help]<br><br>Show the field ONLY if: [abnormal_nbs_experience] = '0'	Do you think having prior experience with NBS would have been helpful in implementing storage disorders?	radio (Matrix), Required<br>1 Not helpful<br>2 A little helpful<br>3 Moderately helpful<br>4 Very helpful
	82	[nbs_exp_factors]<br><br>Show the field ONLY if: [nbs_exp_help] = '2' or [nbs_exp_help] = '3' or [nbs_exp_help] = '4'	What specific factors do you believe contributed to an easier transition?	notes
	83	[nbs_exp_challenge]<br><br>Show the field ONLY if: [nbs_exp_help] = '1' or [nbs_exp_help] = '2' or [nbs_exp_help] = '3' or [nbs_exp_help] = '4'	What did you still find challenging/what were some unexpected challenges you faced?	notes
	84	[addition_of_lysosomal_storage_disorders_to_newborn_complete]	Section Header: *Form Status*<br><br>Complete?	dropdown<br>0 Incomplete<br>1 Unverified<br>2 Complete

Instrument: **Example Survey 2** (example_survey_2)	Enabled as survey	[collapsed]
Instrument: **Example Survey 2** (example_survey_2_e4a8)	Enabled as survey	[collapsed]
Instrument: **Terrell Research Development Project Survey - 2024.2** (terrell_research_development_project_survey_20242)	Enabled as survey	[collapsed]
Instrument: **Terrell Research Development Project Survey - 2024.3** (terrell_research_development_project_survey_20243)	Enabled as survey	[collapsed]
